# Elevated Temperature Differentially Influences Effector-Triggered Immunity Outputs in *Arabidopsis*

**DOI:** 10.3389/fpls.2015.00995

**Published:** 2015-11-09

**Authors:** Alexandra Menna, Dang Nguyen, David S. Guttman, Darrell Desveaux

**Affiliations:** ^1^Department of Cell and Systems Biology, University of Toronto, Toronto, ON, Canada; ^2^Centre for the Analysis of Genome Evolution and Function, University of Toronto, Toronto, ON, Canada

**Keywords:** *Arabidopsis thaliana*, *Pseudomonas syringae*, effector-triggered immunity, hypersensitive response, elevated temperature, abiotic stress, programmed cell death, disease resistance

## Abstract

*Pseudomonas syringae* is a Gram-negative bacterium that infects multiple plant species by manipulating cellular processes via injection of type three secreted effectors (T3SEs) into host cells. Nucleotide-binding leucine-rich repeat (NLR) resistance (R) proteins recognize specific T3SEs and trigger a robust immune response, called effector-triggered immunity (ETI), which limits pathogen proliferation and is often associated with localized programmed cell death, known as the hypersensitive response (HR). In this study, we examine the influence of elevated temperature on two ETI outputs: HR and pathogen virulence suppression. We found that in the *Arabidopsis thaliana* accession Col-0, elevated temperatures suppress the HR, but have minimal influence on ETI-associated *P. syringae* virulence suppression, thereby uncoupling these two ETI responses. We also identify accessions of *Arabidopsis* that exhibit impaired *P. syringae* virulence suppression at elevated temperature, highlighting the natural variation that exists in coping with biotic and abiotic stresses. These results not only reinforce the influence of abiotic factors on plant immunity but also emphasize the importance of carefully documented environmental conditions in studies of plant immunity.

## Introduction

Gram negative bacterial pathogens, such as *Pseudomonas syringae*, use a type III secretion system (T3SS) to inject type III secreted effectors (T3SEs) into host cells where they target cellular components to disrupt host immunity (Lewis et al., 2009; Deslandes and Rivas, 2012). In response, plants have evolved intracellular nucleotide-binding domain leucine-rich repeat (NLR) proteins to directly or indirectly recognize effectors and initiate effector-triggered immunity (ETI; Jones and Dangl, 2006; Dodds and Rathjen, 2010). ETI is often, but not always, associated with a localized programmed cell death (PCD) termed the hypersensitive response (HR; Jones and Dangl, 2006).

Temperature has been shown to modulate outputs of plant ETI in several plant systems. For example, the tobacco *N* (necrosis) gene encodes an NLR that confers resistance to tobacco mosaic virus (TMV) and a rapid HR after infection at a permissive temperature (21°) (Whitham et al., 1994). However, shifting plants to an elevated temperature (28°) prevents the development of N-mediated-HR (Samuel, 1931; Whitham et al., 1994). Similarly, the HR responses induced by the T3SEs AvrRpt2, AvrRpm1, and AvrRps4 in *Arabidopsis thaliana* (hereafter *Arabidopsis*) are inhibited at elevated temperature (28°C) (Goel et al., 2008; Freeman and Beattie, 2009; Wang et al., 2009; Cheng et al., 2013). Furthermore, constitutive expression of the NLR RPS4 results in temperature-conditioned autoimmunity that is suppressed at 28°C (Heidrich et al., 2013). Although these examples demonstrate a strong influence of temperature on the ETI-associated HR, limited information is available about how ETI-associated virulence suppression is affected by temperature.

In our studies of the ETI response activated by the T3SE HopZ1a in *Arabidopsis*, we have observed that at elevated temperatures (28–30°C) the HR induced by this T3SE was suppressed, however, the reduction of bacterial growth associated with this ETI response remained intact. As such, two facets of the ETI-response, HR and virulence suppression can be differentially affected, and uncoupled, by elevated temperature conditions.

## Materials and Methods

### Plant Materials, Growth Conditions, Stress Conditions

*Arabidopsis* plants were grown in 12 h of light (130–150 microeinsteins m^–2^ s^–1^) and 12 h of darkness at 21–22°C and 50–65% humidity in Sunshine Mix 1 soil supplemented with 20:20:20 fertilizer at 1g/L. Except for accession spray assays, all assays were performed in the Col-0 background.

Prior to assays, plants were subjected to temperature priming for 24 h to avoid developmental differences. For elevated temperature priming, plants were incubated in 24 h of light (130–150 microeinsteins m^–2^ s^–1^) at 28–30°C and 15–50% humidity. Conversely, for room temperature control condition, plants were primed in 24 h of ambient light intensity, ambient room temperature (21–24°C), and ambient relative humidity (15–50%). Experiments were all performed at ambient temperature, and plants were placed in respective conditions (elevated or room temperature) for the duration of the experiment.

### *Pseudomonas syringae* Macroscopic HR, Ion Leakage, and *in planta* Bacterial Growth Assays

*Pseudomonas syringae* pv. tomato DC3000 (*Pto*DC3000) carried empty vector (pUCP20), HopZ1a (Ma et al., 2006) or AvrRpt2 (Mudgett and Staskawicz, 1999). For macroscopic HR assays, *P. syringae* was resuspended to an optical density at 600 nm (OD600) of 0.1 (5 × 10^7^ CFU/mL) and syringe-infiltrated into the right side of leaves of 5 week-old wild type Col-0 *Arabidopsis*. Macroscopic HR phenotypes were scored between 12 and 16 hpi. HR phenotypes were scored as “strong” (paper-like dryness and grayish in color) or “weak” (wilting but not dry and retaining green color).

For ion leakage assays, *P. syringae* was resuspended and diluted to a concentration of OD_600_ = 0.04 (2 × 10^7^ CFU/mL), and syringe-infiltrated into four leaves per plant of 4.5–5 week-old wild type Col-0 *Arabidopsis*. After infiltration, four leaf disks (1.5 cm^2^) per plant (one disk per leaf) were harvested, washed in sterile double distilled H_2_O (ddH_2_O) for 30 min on a bench-top shaker at 250 rpm, and transferred to 6 mL of sterile ddH_2_O. Readings were obtained with an Orion 3 Star benchtop conductivity meter (Thermo Fisher Scientific Inc., Fort Collins, CO, USA).

For *in planta* bacterial growth assays, *P. syringae* was resuspended and diluted to obtain a concentration of OD_600_ = 0.0002 (1 × 10^5^ CFU/mL), and infiltrated into four leaves per plant of 3.5–4 week-old wild type Col-0 *Arabidopsis*. On Day 0, four disks (1 cm^2^) per plant (one disk per leaf) were harvested from 2 to 3 plants approximately 1 h after infiltration, ground in 1 mL sterile 10 mM MgCl_2_, and plated on KB with rifampicin (50 μg/mL) and cycloheximide (50 μg/mL) for colony quantification. On Day 3, four disks (1 cm^2^) per plant (one disk per leaf) were harvested from 10 plants, ground in 1 mL sterile 10 mM MgCl_2_, and plated on KB with rifampicin (50 μg/mL) and cycloheximide (50 μg/mL) for colony quantification.

For disease resistance spray assays, *P. syringae* was resuspended and diluted to obtain a concentration of OD_600_ = 0.4 (2 × 10^8^ CFU/mL) or OD_600_ = 0.8 (4 × 10^8^ CFU/mL). Silwet L-77 was added to 0.04% (v/v). Plants were sprayed on Day 0 and Day 3 using a Prevall sprayer, and domed immediately. Dome was removed on Day 4 after second spray, and plants were monitored for disease symptoms up to 7–10 days.

## Results

### Elevated Temperature Suppresses ETI-associated Hypersensitive Responses

**FIGURE 1:**
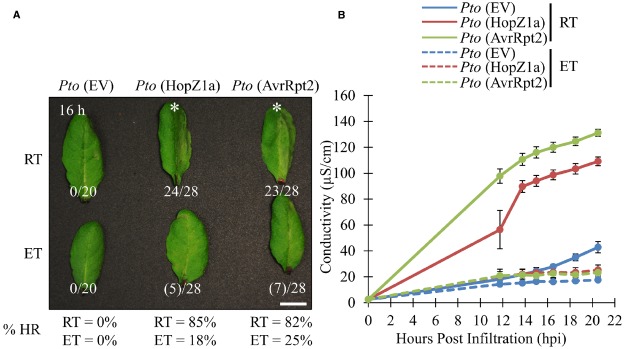
**ETI-associated hypersensitive response and ion leakage are suppressed by elevated temperature in ***Arabidopsis*** accession Col-0.** For ambient room temperature (RT) or elevated temperature (ET) conditions, plants were subject to 21–24°C and 30°C, respectively, for 24 h prior to infiltration. **(A)** *Arabidopsis* half-leaves were infiltrated with *Pto*DC3000 (*Pto*) expressing empty vector (EV), HopZ1a or AvrRpt2 at OD_600_ = 0.1 (5 × 10^7^ CFU/mL). Hypersensitive response (HR) phenotypes were scored between 10 and 20 h post infiltration (hpi). Fractions indicate number of leaves displaying HR phenotype out of total number of leaves infiltrated. This is also reflected in percentages at the bottom of the figure. Numbers in brackets indicate weak HR phenotypes; asterisks (*) indicate strong HR phenotypes on the images presented. Macroscopic HR assays were performed three times with similar results. (RT = 23.7°C, 47% relative humidity (RH); ET = 30.0°C, 48% RH) **(B)** *Arabidopsis* leaves were infiltrated with *Pto*DC3000 (*Pto)* expressing empty vector (EV), HopZ1a or AvrRpt2 at OD_600_ = 0.04 (2 × 10^7^ CFU/mL). Four leaf disks per plant (one disk per leaf, total leaf tissue 1.5 cm^2^) were transferred to 6 mL of sterile ddH_2_O and conductivity readings were taken between 10 and 20 hpi (see Materials and Methods). Ion leakage assays were conducted three times with similar results. (RT = 24.6°C, 51% RH; ET = 30.0°C, 50% RH).

We examined two T3SEs delivered by *P. syringae* pv. *tomato* (*Pto*DC3000), HopZ1a and AvrRpt2, which elicit ETI, and associated HR in *Arabidopsis* accession Col-0 (Bent et al., 1994; Mindrinos et al., 1994; Lewis et al., 2008). We examined the ability of both effectors to induce ETI-associated HR under either ambient (21–24°C) or elevated temperature conditions (30°C). Both groups of plants were grown under identical growth conditions for 3 to 5 weeks, and subject to experimental temperatures 24 h prior to infiltration (see Materials and Methods). Delivery of AvrRpt2 or HopZ1a from the virulent *Pto*DC3000 triggers a strong macroscopic HR between 12 and 16 h post infiltration (hpi) at ambient temperature conditions, as expected (Figure 1A). At elevated temperature, both the *Pto*DC3000 (AvrRpt2) and *Pto*DC3000 (HopZ1a)-induced HR was suppressed, with no leaves showing a strong HR between 12 and 16 hpi, and only ∼20% of leaves showing a weak HR-like collapse (see Materials and Methods; Figure 1A).

To quantify the suppressive effects of elevated temperature on ETI-associated HR, we quantified ion leakage as a proxy for HR. As expected, both *Pto*DC3000 (AvrRpt2) and *Pto*DC3000 (HopZ1a) induced HR-associated ion leakage in ambient temperature conditions by 12 hpi relative to the *Pto*DC3000 empty vector (EV) control (Figure 1B). However, under elevated temperature conditions the HR-associated ion leakage for both *Pto*DC3000 (HopZ1a) and *Pto*DC3000 (AvrRpt2)-induced HR was suppressed, and not significantly different from the *Pto*DC3000 (EV) control (Figure 1B).

### ETI-associated Virulence Suppression is not Inhibited by Elevated Temperatures

**FIGURE 2:**
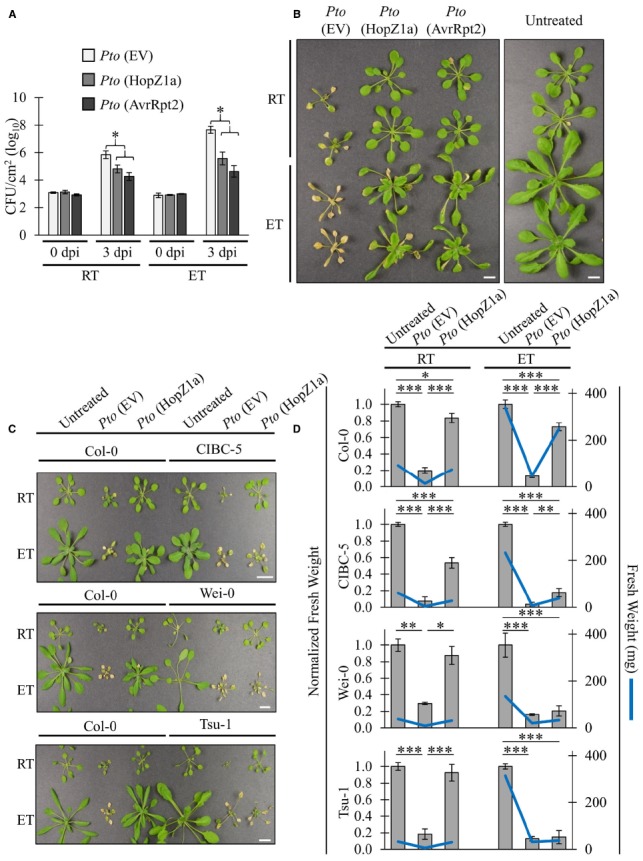
**ETI-associated virulence suppression is not inhibited by elevated temperatures.** For ambient room temperature (RT) or elevated temperature (ET) conditions, plants were subject to 21–24°C and 30°C, respectively, for 24 h prior to infiltration. **(A)** *Arabidopsis* leaves were infiltrated with *Pto*DC3000 (*Pto*) expressing empty vector (EV), HopZ1a, or AvrRpt2 at OD_600_ = 0.0002 (1 × 10^5^ CFU/mL). Bacterial counts were determined 1 h post-infiltration (0 dpi) and 3 days post-infiltration (3 dpi). Two-tailed homoscedastic *t*-tests were performed to test for significant differences. Treatments were compared to empty vector in either the ambient room temperature (RT) or elevated temperature (ET) condition and significant differences are indicated by an asterisk (*P < 0.01). Error bars indicate the standard deviation from the mean of 2 samples (0 dpi) and 10 samples (3 dpi). Bacterial growth assays for HopZ1a and AvrRpt2 were conducted four times with similar results. (RT = 24.6–25.4°C, 16–26% RH; ET = 30.0°C, 25–26% RH) **(B)** *Arabidopsis* plants were spray-inoculated with *Pto*DC3000 (*Pto*) expressing empty vector (EV), HopZ1a or AvrRpt2 at OD_600_ = 0.4 (2 × 10^8^ CFU/mL). Photos shown were taken 10 days post-spraying (dps). Assay was conducted four times with similar results. Scale bar indicates 1 cm. (RT = 22.8–25°C, 48–53% RH; ET = 29.7–30.0°C, 35–50% RH) **(C)** *Arabidopsis* accessions Col-0, CIBC-5, Wei-0 and Tsu-1 were spray inoculated with *Pto*DC3000 (*Pto*) expressing empty vector (EV) or HopZ1a at OD_600_ = 0.8 (4 × 10^8^ CFU/mL). Independent Col-0 control plants were grown on same flat as each indicated accession. Photos shown were taken 10 dps. Assay was conducted four times with similar results. Scale bar indicates 1cm. (RT = 22.8–23.7°C, 48–51% RH; ET = 30.0°C, 41–51% RH) **(D)** Normalized and absolute fresh weight measurements of *Arabidopsis* accessions Col-0, CIBC-5, Wei-0 and Tsu-1 for untreated plants, and plants treated with *Pto* (EV) or HopZ1a at 10 dps as in panel (C). Normalized fresh weight values were calculated relative to the respective untreated controls for each temperature and accession assay to account for the greater general growth observed under ET conditions. Absolute fresh weight in milligrams (mg) is shown on the secondary Y-axis. Data are combined from multiple trials. Error bars indicate standard errors. Two-tailed homoscedastic *t*-tests were performed to test significance and asterisk indicate *P*-values (0.05 > * > 0.01 > ** > 0.001 > ***).

We have demonstrated that the ETI-associated HR induced by *Pto*DC3000 (HopZ1a) and *Pto*DC3000 (AvrRpt2) is suppressed in elevated temperature (30°C) conditions, corroborating previously published data examining the influence of elevated temperature on HR (Figure 1; Freeman and Beattie, 2009; Cheng et al., 2013). In order to examine whether ETI-associated virulence suppression was also impaired by elevated temperature incubation, we conducted *in planta* bacterial growth assays (Figure 2A). As expected, the ETI triggered by both T3SEs decreased bacterial growth by ∼1.5 log CFU/cm^2^ at ambient temperature relative to the EV negative control (Figure 2A). Plants incubated at elevated temperature (30°C) exhibited increased bacterial growth of the EV control (∼1.5 log) relative to ambient temperature. Nevertheless, the ETI-associated virulence suppression induced by both T3SEs was still observed under elevated temperature conditions (Figure 2A). In fact, the magnitude of ETI-associated *Pto*DC3000 growth reduction was an order of magnitude greater for both T3SEs at elevated temperatures (∼2.5 log CFU/cm^2^) than at ambient temperature (∼1.5 log CFU/cm^2^; Figure 2A).

We performed disease resistance spray assays in order to qualitatively visualize the immune response at elevated temperatures (Figure 2B). Plants pre-incubated in ambient or elevated temperature were sprayed at a high bacterial density and disease symptoms were monitored. At ambient temperature, disease symptoms and chlorosis of plants sprayed with *Pto*DC3000 (EV) became visible at approximately 2 to 3 days post infection (dpi). At 5 dpi, disease-associated chlorosis became more severe, and plant growth was stunted relative to healthy plants. In contrast, plants sprayed with *Pto*DC3000 (HopZ1a) or *Pto*DC3000 (AvrRpt2) remained relatively healthy, and plant growth resembled uninfected plants (Figure 2B). Similarly, at elevated temperature, plants sprayed with *Pto*DC3000 (HopZ1a) or *Pto*DC3000 (AvrRpt2) remained relatively symptom-free relative to plants infected with *Pto*DC3000 (EV; Figure 2B). This observation combined with the quantification of *in planta* bacterial growth indicated that ETI-associated virulence suppression remained intact under elevated temperature conditions that suppressed ETI-associated HR.

### *Arabidopsis* Accessions Exhibit Varying Temperature Sensitivity

We demonstrated that ETI-associated virulence suppression remains intact under elevated temperature conditions that otherwise suppress ETI-associated HR in the *Arabidopsis* Col-0 accession. In order to examine whether local adaptation to varying climates can influence disease resistance under elevated temperature conditions, we tested *Arabidopsis* accessions isolated from varying geographic climate regions for loss of *Pto*DC3000 (HopZ1a)-triggered immunity at elevated temperature. Although most accessions showed a Col-0 like retention of ETI at elevated temperature (data not shown), we identified three accessions CIBC-5, Wei-0 and Tsu-1, that displayed a loss of ETI-associated virulence suppression at elevated temperature, despite showing a normal (Col-0 like) response at ambient temperature (Figure 2C). Unlike Col-0, CIBC-5, Wei-0 and Tsu-1, developed a chlorotic, disease phenotype when sprayed with *Pto*DC3000 (HopZ1a) in elevated temperature, similar to that of *Pto*DC3000 (EV; Figure 2C).

As we were conducting our spray assays we noted a noticeable size difference in resistant (green) versus susceptible (chlorotic) plants. Therefore, in order to quantify the loss of ETI-associated virulence suppression, we measured the fresh weight (both absolute and normalized) of plants at 10 days post-infection (Figure 2D). We normalized the fresh weight to assess the magnitude of differences between treatment in light of the overall increased growth associated with elevated temperatures. This normalization was done based on the weight of the untreated plant for each accession-by-temperature comparison, and was done independently for each trial. At both ambient and elevated temperature, Col-0 plants sprayed with *Pto*DC3000 (HopZ1a) were of a significantly higher fresh weight than plants sprayed with *Pto*DC3000 (EV). CIBC-5, Wei-0 and Tsu-1 plants sprayed with *Pto*DC3000 (HopZ1a) also had higher fresh weight relative to *Pto*DC3000 (EV) at ambient temperature, but this difference was reduced (CIBC-5) or lost (Wei-0 and Tsu-1) at elevated temperature (Figure 2D). Overall, these data demonstrate that the ETI response of *Arabidopsis* accessions is differentially affected by temperature.

## Discussion

Our study corroborates previous findings that temperature is a direct modulator of plant immunity. However, we find that different outputs of immunity are differentially sensitive to elevated temperature. A previous study has proposed that lower temperatures favor the activation of ETI, whereas ETI is compromised at elevated temperatures (Cheng et al., 2013). In light of our results, we would add that different facets of ETI are differentially influenced by temperature, with HR being more sensitive than ETI-associated virulence suppression. In addition, our results support the hypothesis that HR cell death is dispensable for resistance to *Pto*DC3000.

In order to minimize plant developmental differences between experiments conducted at elevated temperature versus ambient temperature, plants were grown for 3–5 weeks under our standard plant growth conditions, and then acclimated to elevated temperature conditions for 24 h prior to infection assays. Relative humidity was controlled to be within a moderate to low range (15–50%) in order to avoid the additive suppressive effects of high humidity since, like elevated temperature, high relative humidity has also been demonstrated to suppress ETI responses (Samuel, 1931; Jambunathan et al., 2001; Yoshioka et al., 2001; Lorrain et al., 2003; Zhou et al., 2004; Noutoshi et al., 2005; Moeder and Yoshioka, 2008; Freeman and Beattie, 2009; Mosher et al., 2010; Beattie, 2011; Hangyang et al., 2015). Corroborating this data, we have found HopZ1a and AvrRpt2 HR to be delayed under high relative humidity conditions (data not shown). It should be noted that relative humidity was significantly influenced by seasonal variation (i.e., significantly lower in the winter months than in summer months) which contributed to the relatively large humidity range of our experiments despite our control efforts. We nevertheless aimed to maintain humidity below 50% in all cases and to have comparable relative humidity across ambient and elevated temperature treatments (see Figure Legends for specific values).

It is important to note that the suppressed HR at elevated temperature is not due to inhibition of the type III secretion system since *P. syringae* type III effector expression assays are routinely conducted at 28–30°C. Additionally, the *in planta* growth rate of virulent *Pto*DC3000 actually increased at elevated temperature, corroborating previously published data (Freeman and Beattie, 2009; Wang et al., 2009; Cheng et al., 2013).

Two different assays to monitor the ETI-associated HR yielded slightly differing results. Macroscopic HR was significantly weakened in our experiments, but not entirely inhibited (Figure 1A). On the other hand, HR-associated ion leakage was entirely inhibited for both T3SEs tested resulting in measurements similar to those of the EV control (Figure 1B). This is likely influenced by the fact that for ion leakage assays, infiltrated leaf disks are placed in water (i.e., 100% humidity) immediately post-infiltration in order to monitor changes in water conductance. As such, the ion leakage assays are conducted in both an elevated temperature and high humidity environment. We believe that the combined suppressive effects of elevated temperature and humidity result in a complete loss of HR ion leakage associated, whereas macroscopic HR is delayed but still observed since these assays are conducted at ambient relative humidity (Samuel, 1931; Jambunathan et al., 2001; Yoshioka et al., 2001; Lorrain et al., 2003; Zhou et al., 2004; Noutoshi et al., 2005; Moeder and Yoshioka, 2008; Freeman and Beattie, 2009; Mosher et al., 2010; Beattie, 2011; Hangyang et al., 2015).

Our results indicate that elevated temperatures can uncouple the HR response from ETI-associated virulence suppression. This is reminiscent of ETI examples that occur without apparent PCD. The NLRs TAO1 and RPS6 activate ETI in response to the T3SEs AvrB and HopA1 without manifestation of an HR (Shimizu et al., 2003; Gassmann, 2005; Eitas et al., 2008; Naito et al., 2008; Kim et al., 2009). The *Arabidopsis* NLR gene pair RPS4 and RRS1 confer resistance to *Pto*DC3000 expressing AvrRps4, which is accompanied by PCD in Ws-0, but not in Col-0 background (Hinsch and Staskawicz, 1996; Gassmann, 2005; Heidrich et al., 2013). These examples corroborate our observations that HR-associated PCD can be uncoupled from virulence suppression and suggest that HR-associated cell death is not required for resistance against virulent *P. syringae*.

NLRs can be subdivided into two main categories based on their N-terminal domains; coiled coil (CC)-NLR and Toll/interleukin-1 receptor (TIR)-NLR. HopZ1a and AvrRpt2 are recognized by the CC-NLRs ZAR1 and RPS2, respectively (Bent et al., 1994; Mindrinos et al., 1994; Lewis et al., 2010). Most examples of temperature sensitive ETI-responses are mediated by TIR-NLRs suggesting that this NLR subgroup could potentially be more sensitive to elevated temperatures (Hua, 2014). However, our preliminary experiments indicate that AvrRps4-induced virulence suppression, mediated by the TIR-NLRs RPS4 and RRS1, is retained in our elevated temperature conditions (data not shown).

In order to test the influence of local adaptation on ETI-associated virulence suppression, various *Arabidopsis* accessions were tested using spray assays and we identified three accessions that displayed compromised ETI at elevated temperature. CIBC-5 was isolated from Ascot, Berkshire, United Kingdom (51.4084°N, 0.6707°W), Tsu-1 from Tsushima, Japan (34.202°N, 129.29°E) and Wei-0 from Weiningen, Switzerland (47.419°N, 8.4326°E). The Col-0 accession which retained ETI-virulence suppression at elevated temperature is most similar to the Gü-0 accession, originally isolated from Gückingen, Germany (50.391°N, 8.008°E) and propagated for research use in the mid-1900s in Columbia, Missouri, USA (38.952°N, 92.334°W) (Nordborg et al., 2005). The CIBC-5 and Wei-0 accessions were isolated from locations that would experience slightly cooler average temperatures (highest monthly summer average temperature = 23°and 24°, respectively) than Gü-0 (25°), which may explain the lower tolerance of their ETI responses. However, Tsu-1 (33°) was isolated from a location that experiences much higher temperatures than any of the other accession discussed here (worldweatheronline.com). Therefore, the differences observed in the temperature tolerance of their respective ETI responses is likely influenced by additional factors. Nevertheless, the ecotypic differences observed in ETI responses at elevated temperature indicate that temperature tolerance may be an adapted trait that should be genetically tractable.

In this study, we have demonstrated that the virulence suppression and HR outputs of ETI are differentially sensitive to elevated temperature. At elevated temperatures, HR is significantly delayed or inhibited depending on the assay method used, whereas immunity, measured as bacterial growth inhibition or disease symptom development, is relatively unaffected. These observations not only emphasize the importance of carefully documented plant growth conditions in studies of plant immunity, but also highlight the differential sensitivity of various immunity outputs to abiotic stresses.

### Conflict of Interest Statement

The authors declare that the research was conducted in the absence of any commercial or financial relationships that could be construed as a potential conflict of interest.
